# Colony phase variation switch modulates antimicrobial tolerance and biofilm formation in *Acinetobacter baumannii*

**DOI:** 10.1128/spectrum.02956-23

**Published:** 2024-01-11

**Authors:** Fizza Mushtaq, Aftab Nadeem, Abdelbasset Yabrag, Anju Bala, Nabil Karah, Nikola Zlatkov, Sun Nyunt Wai, Bernt Eric Uhlin, Irfan Ahmad

**Affiliations:** 1 Department of Molecular Biology and Umeå Centre for Microbial Research (UCMR), Umeå University, Umeå, Sweden; 2 Institute of Biomedical and Allied Health Sciences, University of Health Sciences, Lahore, Pakistan; 3 The Laboratory for Molecular Infection Medicine Sweden (MIMS), Umeå University, Umeå, Sweden; 4 Department of Microbiology, Tumor and Cell Biology, Karolinska Institute, Stockholm, Sweden; Dominican University New York, Orangeburg, New York, USA

**Keywords:** opaque colony, translucent colony, colisitin

## Abstract

Carbapenem-resistant *Acinetobacter baumannii* causes one of the most difficult-to-treat nosocomial infections. Polycationic drugs like polymyxin B or colistin and tetracycline drugs such as doxycycline or minocycline are commonly used to treat infections caused by carbapenem-resistant *A. baumannii*. Here, we show that a subpopulation of cells associated with the opaque/translucent colony phase variation by *A. baumannii* AB5075 displays differential tolerance to subinhibitory concentrations of colistin and tetracycline. Using a variety of microscopic techniques, we demonstrate that extracellular polysaccharide moieties mediate colistin tolerance to opaque *A. baumannii* at single-cell level and that mushroom-shaped biofilm structures protect opaque bacteria at the community level. The colony switch phenotype is found to alter several traits of *A. baumannii*, including long-term survival under desiccation, tolerance to ethanol, competition with *Escherichia coli*, and intracellular survival in the environmental model host *Acanthamoeba castellanii*. Additionally, our findings suggest that extracellular DNA associated with membrane vesicles can promote colony switching in a DNA recombinase-dependent manner.

**IMPORTANCE:**

As a WHO top-priority drug-resistant microbe, *Acinetobacter baumannii* significantly contributes to hospital-associated infections worldwide. One particularly intriguing aspect is its ability to reversibly switch its colony morphotype on agar plates, which has been remarkably underexplored. In this study, we employed various microscopic techniques and phenotypic assays to investigate the colony phase variation switch under different clinically and environmentally relevant conditions. Our findings reveal that the presence of a poly N-acetylglucosamine-positive extracellular matrix layer contributes to the protection of bacteria from the bactericidal effects of colistin. Furthermore, we provide intriguing insights into the multicellular lifestyle of *A. baumannii*, specifically in the context of colony switch variation within its predatory host, *Acanthamoeba castellanii*.

## INTRODUCTION

*Acinetobacter baumannii* has recently emerged as one of the most prevalent causes of multidrug-resistant nosocomial infections (1). In a classical study conducted in 1951, a clinical strain of *A. baumannii* then known as *Bacterium anitratum* exhibited heterogeneity in colony morphology on infusion agar plates (2). *B. anitratum* produced two distinct forms of colonies on agar plates. The mucoid colonies appeared as opaque, dome-shaped, and glistering white, whereas non-mucoid colonies were shown as translucent and grey or blue based on the reflection of transmitted light (2). Recently, a similar colony morphotype switch based on the transmission of incident light has been thoroughly characterized (3–5). The opaque and translucent colonies are unstable and interconvertible at high frequency in a cell density-dependent manner. In relation to colony variation, several phenotypic changes have been observed, such as altered cell morphology, surface motility, biofilm formation, antibiotic resistance, and virulence (3, 5). The increased resistance of virulent opaque variants to hospital disinfectants and desiccation plays a significant role in the environmental persistence and epidemic spread of disease. In a mouse model, the opaque colony population becomes hypervirulent and possesses thicker capsules as compared to its translucent counterparts (3). The expression of TetR-type transcriptional regulators (TTTRs) redundantly drives the switching of opaque phenotype to translucent cells. There are 11 structurally related TTTRs identified in the genome of *A. baumannii*. To inhibit TTTRs associated colony switch drive, the deletion of at least four of these genes is required (6).

We have recently analyzed colony phase variation switch in several clinical isolates of *A. baumannii* (7). Several phenotypic traits such as biofilm formation, production of outer membrane vesicles (MVs), and virulence in *Caenorhabditis elegans*, were significantly altered upon colony phase variation switch in *A. baumannii* clinical isolates. The degree of opacity and switching frequency varied among isolates (7). Here, we investigate the fitness benefits and regulation of colony switch phase variation in *A. baumannii*.

## RESULTS

### Opaque colony morphotype is associated with the presence of WGA positive extracellular polysaccharide layer on the surface of opaque colonies

**Fig 1:**
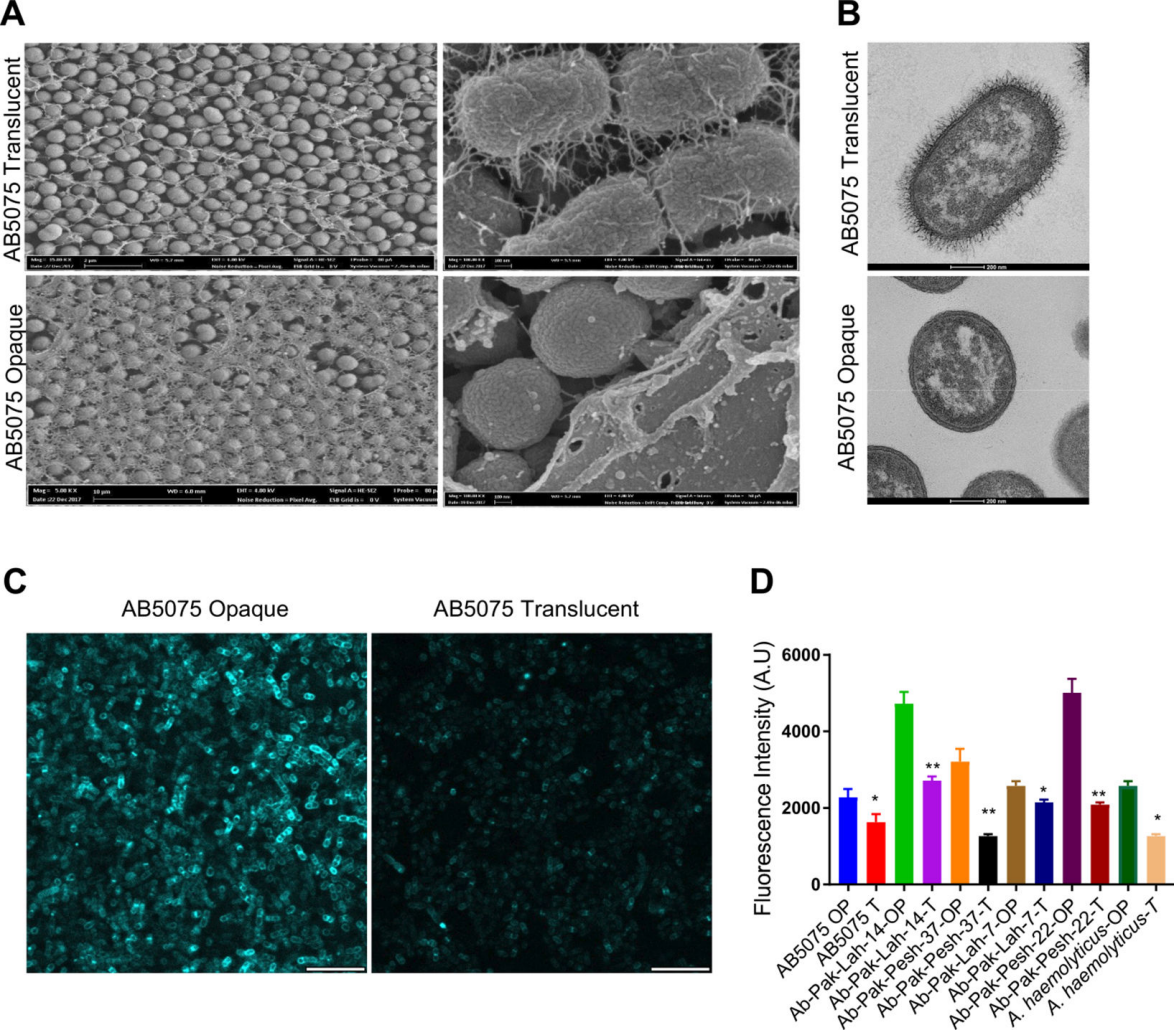
Opaque colonies express extracellular polysaccharide moieties. (**A**) Left panels: Scanning electron microscopy visualization of bacterial cells from opaque and translucent colonies of *A. baumannii* AB5075. Right panels: Higher magnification images to illustrate the presence of an additional layer of extracellular material in case of opaque colonies. (**B**) Transmission electron microscopy of individual cells from opaque and translucent colonies of *A. baumannii* AB5075. (**C**) Representative confocal laser scanning microscopy images of WGA-labeled opaque and translucent colonies of *A. baumannii* AB5075. Scale bars = 5 µm. (**D**) Fluorescence quantification of samples from pair-wise opaque and translucent variants of different *A. baumannii* clinical isolates and *A. haemolyticus* upon labeling with Aelxa647-WGA.

To examine the surface components of *A. baumannii* that influence the opaque or translucent appearance of colonies, scanning electron microscopy (SEM) was performed on opaque and translucent colonies of the hypervirulent strain *A. baumannii* AB5075. For this purpose, colonies were fixed directly on agar plates before processing the samples for SEM visualization. The SEM imaging of cells from opaque and translucent colonies revealed that bacteria grown within opaque colonies were covered by an extracellular matrix layer, whereas no such layer was observed on the surface of cells from translucent colonies (Fig. 1A). In contrast, bacteria growing in translucent colonies express abundant extracellular pili. Similar pilus-like structures were also visible under transmission electron microscopy upon negative staining of translucent colony-forming cells (Fig. 1B). The presence of an extracellular matrix around opaque colony forming cells prompted us to investigate the nature of this extracellular matrix. The binding of fluorescent dye-conjugated wheat germ agglutinin (WGA) to the surfaces of opaque and translucent colonies was examined. WGA is a carbohydrate-binding lectin with a high affinity for N-acetylglucosamine moieties in glycoproteins of *A. baumannii* (8). Confocal laser scanning microscopy (CLSM) of WGA-labeled bacteria revealed that WGA could bind more efficiently to the surface of bacteria from opaque colonies as compared to bacteria from translucent colonies (Fig. 1C). To investigate whether WGA-binding carbohydrate moieties are produced more abundantly in particular by bacteria forming opaque colonies, as in the case of other strains of *A. baumannii* and *Acinetobacter haemolyticus*, pairwise opaque and translucent colony variants of different clinical isolates were monitored for WGA binding in 96-well microtiter plates after growth overnight. The WGA fluorescence labeling of opaque variants was substantially greater than that of translucent variants for all tested clinical isolates (Fig. 1D). Taken together, these findings and SEM image analyses suggest that the opaque colony morphotype is associated with the presence of extracellular carbohydrate moieties that surround the bacterial cells forming opaque colonies.

### WGA-binding extracellular moieties protect bacteria from the bactericidal effect of colistin

**Fig 2:**
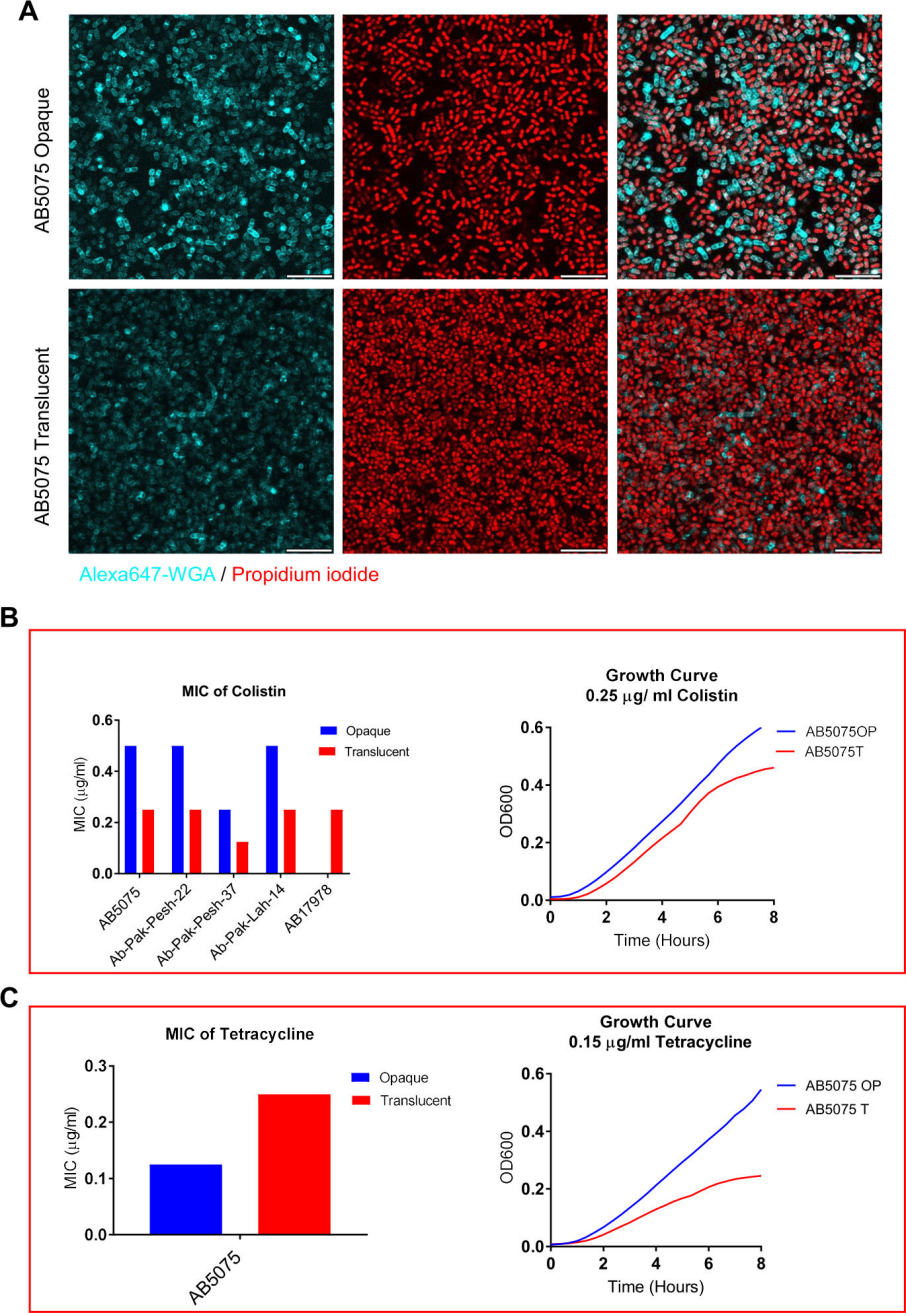
WGA-binding extracellular moieties protect opaque *A. baumanni* from colistin. (**A**) Confocal laser microscopy images of opaque and translucent variants of *A. baumannii* AB5075 grown in an 18-well glass chamber upon the treatment of colistin. Propidium iodide was used to differentiate dead and live bacteria upon the treatment with colistin. Red: PI-positive cells (dead cells), Blue: WGA binding representing the presence of extracellular polysaccharide moieties. Scale bars = 5 µm. (**B**) Left panel: Minimum inhibitory concentration of colistin in opaque and translucent colony variants of *A. baumannii* strain AB5075 as determined by microbroth dilution method. Right panel: Growth curves of opaque and translucent *A. baumannii* AB5075 grown in a microtiter plate in LB medium with subinhibitory concentration of colistin at 37°C using the built-in temperature control mode of the Spark multimode plate reader (Tecan). *Y*-axis represents the optical density (OD_600_) measured with an interval of 20 min. The experiment was done in triplicate, and the curves were drawn using the mean OD_600_ values. (**C**) Left panel: Minimum inhibitory concentration of tetracycline in opaque and translucent colony variants of *A. baumannii* AB5075 as determined by microbroth dilution method. Right panel: Growth curves of opaque and translucent *A. baumannii* AB5075 grown in a microtiter plate in LB medium with subinhibitory concentration of tetracycline at 37°C using the built-in temperature control mode of the Spark multimode plate reader (Tecan). *Y*-axis represents the optical density (OD_600_) measured with an interval of 20 min. The experiment was done in triplicate, and the curves were drawn using the mean OD_600_ values.

We were further interested in investigating if the polysaccharide layer formed during the opaque lifestyle of bacterial growth might play a role in the antimicrobial tolerance of the bacteria. The polycationic drugs polymyxin B and colistin (polymyxin E) are among the most selected options to treat infections of carbapenem-resistant *A. baumannii* (9, 10). Since opaque variants of *A. baumaannii* harbor WGA-binding extracellular moieties (Fig. 1C and D), such components were considered to have a potential role in mediating colistin tolerance. Therefore, the impact of these polysaccharide moieties on mediating colistin tolerance at the single-cell level was investigated. For that, *A. baumannii* grown in an 18-well glass plate was treated with colistin and subsequently labeled with WGA and propidium iodide (PI), the latter to monitor the appearance of dead bacterial cells. The CLSM imaging revealed that colistin-mediated bacterial killing occurred only in WGA-negative cells (Fig. 2A). This finding suggests that WGA-binding polysaccharide moieties protected opaque variants from the bactericidal effect of colistin at the single-cell level.

Then, we compared opaque and translucent variants of *A. baumannii* clinical isolates for susceptibility to colistin using the microbroth dilution method. The MIC values for colistin were found to be two times higher in the case of opaque variants in comparison with their translucent counterparts of the tested clinical isolates (Fig. 2B). Furthermore, in the presence of a subinhibitory concentration of colistin, there was a somewhat slower growth of the translucent variant of strain AB5075 (Fig. 2B). To investigate whether the effect of colony switching on antimicrobial drug tolerance would be a specific feature occurring with colistin, we compared the MIC of tetracycline between the opaque and translucent variants of strain AB5075. When the two colony variants of strain AB5075 were preliminarily tested on LB agar plates for growth in the presence of tetracycline, we observed that the translucent variant appeared to tolerate this antibiotic better (Fig. S1). Moreover, in contrast to the MIC value for colistin, we observed a twofold higher MIC of tetracycline in the case of the translucent AB5075 variant when compared to the opaque variant (Fig. 2C). These findings suggest that the colony phase variation switch mediates differential alterations in tolerance to colistin and tetracycline in LB broth culture. However, also in the presence of a subinhibitory concentration of tetracycline there was slower growth of the translucent *A. baumannii* (Fig. 2C).

### Formation of mushroom-shaped biofilm structures additionally protect opaque colonies from colistin in an artificial urine medium

**Fig 3:**
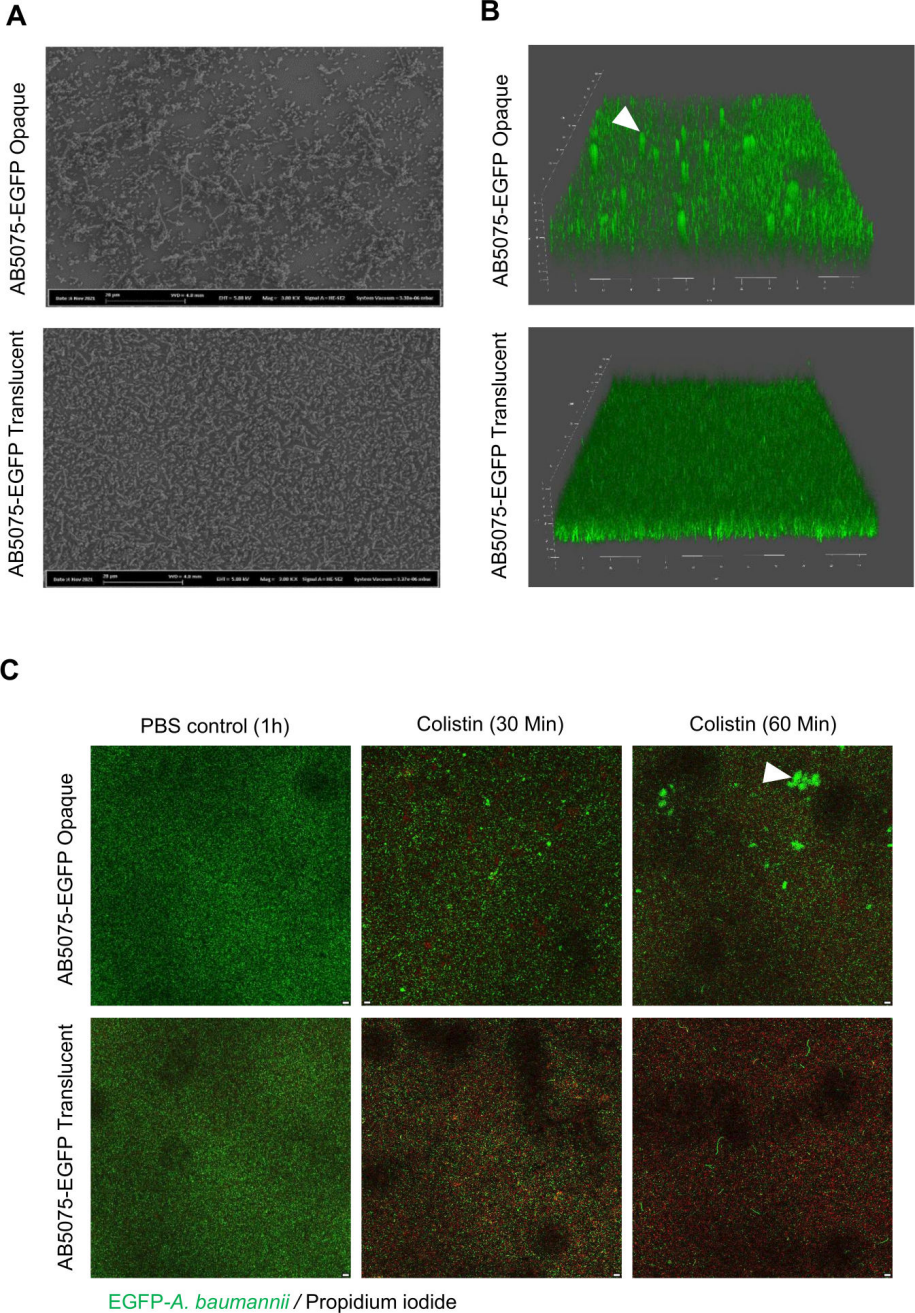
3D biofilm structures formed by opaque and translucent *A. baumannii* AB5075. (**A**) Scanning electron microscopy visualization of bacteria from *A. baumannii* AB5075 grown in the pellicle formed by the opaque and translucent colony morphotype, respectively. (**B**) 3D live cell confocal laser microscopy images of biofilms formed by *A. baumannii* AB5075 opaque and translucent variants expressing green fluorescent protein within static flow cells after 72 h of incubation at 30°C. White arrowhead indicates dense patches of biofilm in opaque variant of *A. baumannii*. (**C**) Live cell confocal microscopy image of opaque and translucent variants of *A. baumannii* AB5075 expressing green fluorescent protein after growth in static flow cells and upon 30 or 60 min treatment with 1 µg/mL colistin. Propidium iodide was used to differentiate dead and live bacteria upon the treatment with colistin. Live cells are shown in green and dead cells are shown in red. Dense patches of biofilm in opaque colonies (as indicated by a white arrowhead) seemed protected from the bactericidal effect of colistin. Scale bars = 5 µm.

Using artificial urine as a growth medium, biofilm formation was then analyzed to mimic clinical growth conditions. For that purpose, variants of *A. baumannii* with constitutive expression of green fluorescent protein were grown in artificial urine medium (AUM) (11) in static glass chambers. SEM was used to visualize biofilm structures within the pellicle formed at air-liquid interphase, and live cell confocal laser microscopy was used to visualize biofilms formed within the glass chambers. The visualization of the pellicle matrix by SEM revealed that the pellicle of the opaque variant contained extracellular matrix components and unevenly distributed clumps of biofilm, whereas the pellicle of the translucent variant merely contained spatially arranged cells (Fig. 3A).

A 3D visualization of biofilm formed by *A. baumannii* revealed that the opaque variant formed mushroom-like biofilm structures when grown in artificial urine medium, whereas the translucent variant formed a uniformly distributed layer of biofilm without visible mushroom-shaped structures (Fig. 3B). The formation of Csu pili-dependent mushroom-shaped biofilm structures has been reported for *A. baumannii* strain 17978 when grown in rotating biofilm bioreactors (12).

The bactericidal effect of colistin was also tested with static cultures in glass chambers. EGFP labeled bacterial strains were grown for 48 h within the ibidi glass chambers and treated with colistin (1 µg/mL). PI was used to detect cells that lost viability upon colistin treatment. Live cell confocal laser microscopic images revealed that the number of PI positive cells (dead cells) was higher in translucent *A. baumannii* AB5075 as compared to its opaque counterpart (Fig. 3C). More specifically, the cells within mushroom-shaped structures were not affected by the treatment with colistin. This finding suggests that the opaque variant exhibits a fitness advantage over its translucent counterpart and tolerates colistin through the formation of biofilm patches.

### Opaque variants exhibit fitness advantage over translucent counterparts in several environmental settings

**Fig 4:**
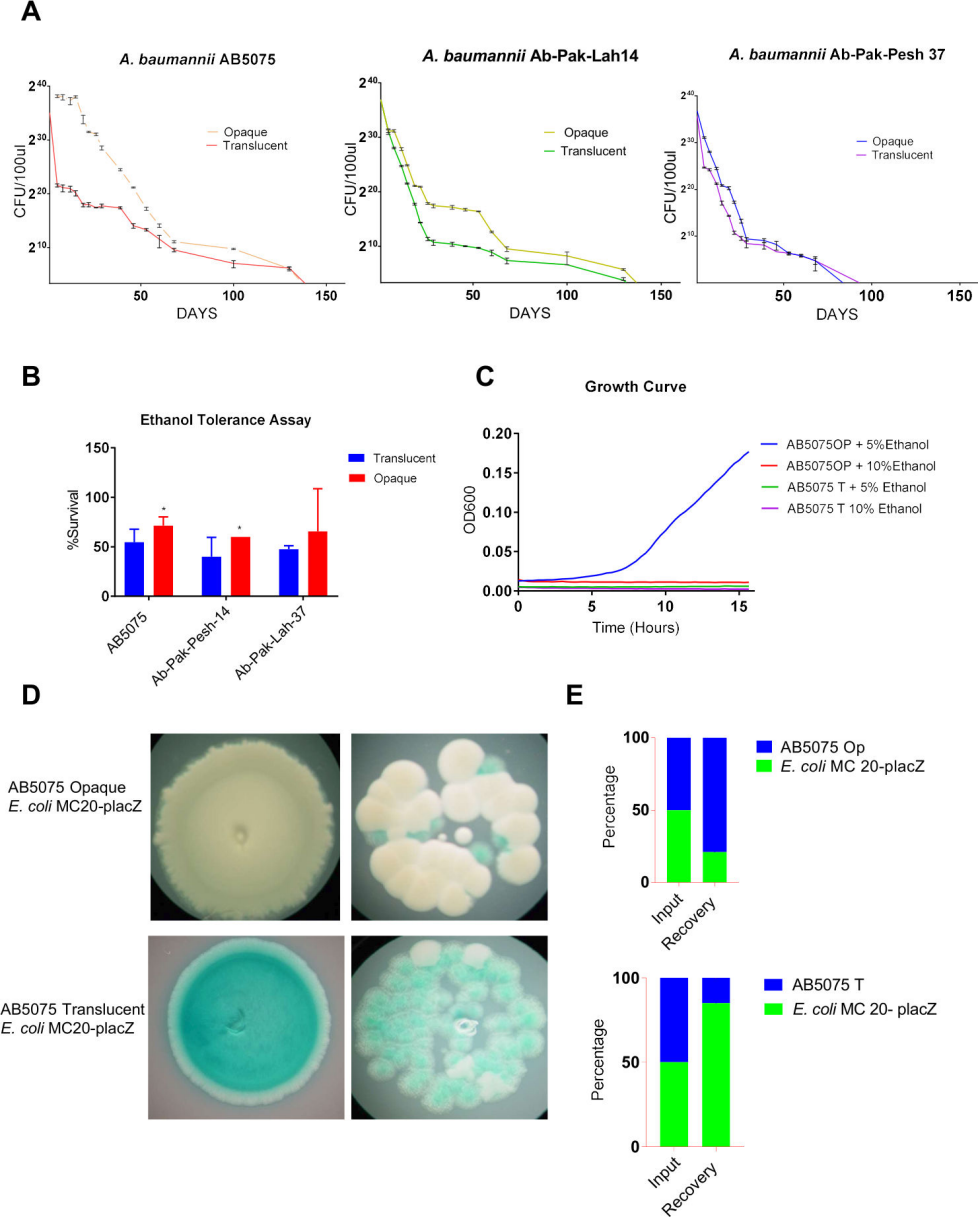
Comparative analysis of opaque and translucent variants of *A. baumannii* for survival under various stress-inducing conditions. (**A**) The survival curves illustrate the number of bacteria that remained viable in samples representing opaque and translucent variants of clinical isolates under desiccation for the period of up to 150 days. (**B**) Ethanol tolerance test estimating the survival of opaque and translucent variants of *A. baumannii* isolates upon the treatment with 5% ethanol in PBS at room temperature for 4 h. (**C**) Growth curves of opaque and translucent *A. baumannii* AB5075 grown in a microtiter plate in LB medium supplemented with 5% or 10% ethanol. The experiment was performed at 37°C using the built-in temperature control mode of the Spark multimode plate reader (Tecan). The *Y*-axis represents the optical density (OD_600_) measured with an interval of 20 min for 16 h. The curves represent the mean OD_600_ values from experiment done in triplicate. (**D**) Photographic images of the mixed colonies of *A. baumannii* and *E. coli* on LB agar plates supplemented with carbenicillin, IPTG, and X-gal. White spots represent *A. baumannii* and blue spots represent *E. coli* MC 20; Left panel: initial inoculum consists of 10^6^ bacteria (1:1 ratio of *A. baumannii* and *E. coli*); Right panel: initial inoculum consists of 10^3^ bacteria (1:1 ratio of *A. baumannii* and *E. coli*). (**E**) A bar chart diagram compares the number of *E. coli* MC 20 and *A. baumannii* variants upon growing together on agar plates.

**Fig 5:**
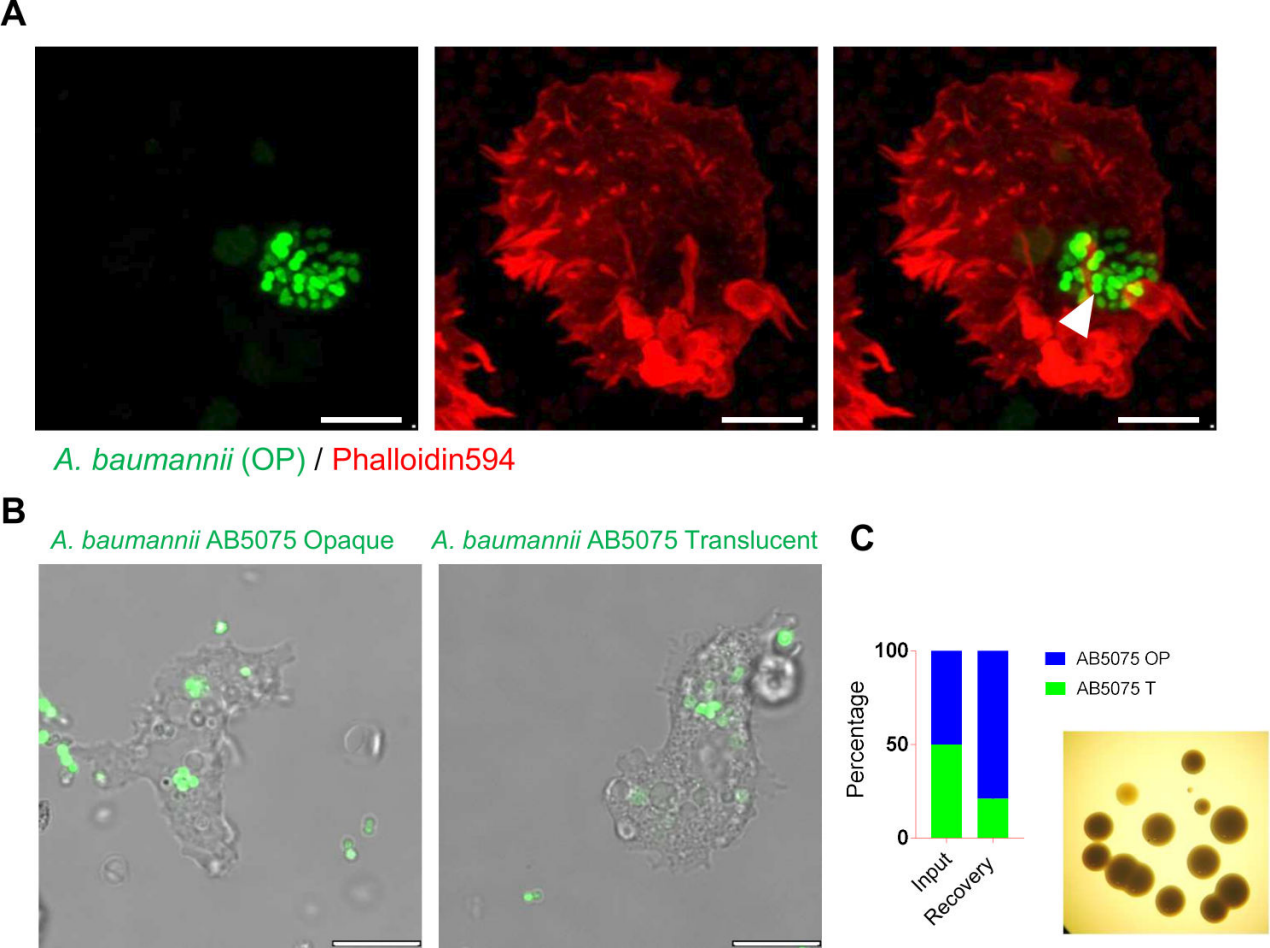
Interaction of *A. baumannii* AB5075 with its environmental host *A. castellanii*. (**A**) Confocal microscopic visualization of *Acanthamoeba castellanii* harboring intracellular *A. baumannii* 48 h after infection. The white arrowhead indicates intracellular localization of EGFP-*A. baumannii* (green). *A. castellanii* was stained with actin marker, Phalloidin594 (red). Scale bars = 5 µm. (**B**) Confocal microscopic visualization of phalloidin labeled (Actin) *A. castellanii* harboring intracellular opaque *A. baumannii* to illustrate localization of intracellular dividing bacteria within a distinct vacuole. Scale bars = 10 µm. (**C**) Percent recovery of *A. baumannii* AB5075 variants after mixed infection of opaque and translucent *A. baumannii* with 1:1 ratio.

The opaque phenotype appeared important for the virulence of *A. baumannii*. However*,* very little is known about its role in different environmental settings. Therefore, we compared opaque and translucent variants of clinical isolates for fitness in several environmental settings, including the ability to survive on a plastic surface under long-term desiccation for up to 150 days, the ability to tolerate a subinhibitory concentration of ethanol, and the ability to compete with other bacterial species. The loss of viability under long-term desiccation appeared to occur in two phases as identified by the periodic CFU measurements of sampled bacteria (Fig. 4). In the first phase, the number of surviving bacteria decreased exponentially over time, whereas the bacteria in the second phase were apparently more persistent. The CFU ability of translucent variants was distinctly more decreased than that of the opaque variants during the first phase as indicated by survival curves for all three tested isolates. Altogether, these findings suggest that the opaque colony morphotype has higher fitness to cope with long-term desiccation, while there was little or no apparent difference in the appearance of persistent cells (second phase of survival curve) between the two variants. In addition, the tolerance of opaque and translucent variants to short-term desiccation under additional stress was compared. Phosphate-buffered saline (PBS) containing 5% ethanol was used to induce stress for this purpose. The number of viable bacteria, as determined by the CFU count upon treatment with 5% ethanol, was consistently lower for the translucent variants when compared to their opaque counterparts (Fig. 4B). Furthermore, 5% ethanol abolished the growth of translucent *A. baumannii*, whereas opaque *A. baumannii* could grow in the presence of this concentration of ethanol (Fig. 5C). However, neither variant did grow in the presence of 10% ethanol. These findings suggest that opacity might have some role in the protection of *A. baumannii* from membrane-destabilizing agents, as here tested with ethanol.

In several environmental and clinical circumstances, different bacterial populations may compete to establish their persistence. To investigate the possible impact of colony phase variation switching of *A. baumannii* in competition with other bacterial species, *Escherichia coli* was used as a model organism for testing interbacterial species competition. For that, *E. coli* MC20 harboring the *lacZ^+^* on a plasmid was examined for its ability to compete with AB5075. LacZ expression was used to distinguish *E. coli* from *A. baumannii* through the production of blue colonies on LB agar plates supplemented with IPTG and X-gal. On agar plates, *E. coli* and *A. baumannii* cells were spotted at a ratio of 1:1, and after 24 h of co-incubation, the ratio of viable *A. baumannii* and *E. coli* was estimated by counting CFUs of viable bacteria. The results revealed that opaque *A. baumannii* inhibited the growth of *E. coli* (Fig. 4D and E). However, surprisingly, translucent *A. baumannii* did not diminish the growth of *E. coli*. Instead, *E. coli* outcompeted translucent *A. baumannii* (Fig. 4D and E). This finding suggests that translucent *A. baumannii* may be less fit to survive in competition with other bacterial species, as shown with *E. coli*. Altogether, these findings suggest that the opaque variant of *A. baumannii* exhibits a fitness advantage over its translucent variant in several environmentally relevant stress conditions, such as short-term desiccation, exposure to ethanol, and competing with *E. coli*.

### *A. baumannii* resides as multicellular bacterial communities within a vacuole of the environmental predator *Acanthamoeba castellannii*, where the opaque variant outcompetes its translucent counterpart

Next, we examined the ability of AB5075 opaque and translucent variants expressing EGFP to survive and replicate in *Acanthamoeba castellanii*, an environmental predatory host. Both variants of *A. baumannii* were found capable of residing intracellularly in *A. castellanii* (Fig. S2A). Confocal microscopic imaging of intracellular bacteria revealed that *A. baumannii* resides intracellularly within a distinct vacuole in the form of multicellular bacterial communities (Fig. 5A and B; Fig.S2B). Subsequently, we infected *A. castellanii* simultaneously with opaque and translucent bacteria in a competition experiment to assess fitness advantage within intracellular multicellular communities. As described in the Materials and Methods, the number of intra-amoeba bacteria was estimated. The data suggest that the number of opaque colony-forming AB5075 was two- to fourfold higher than that of its translucent counterpart. (Fig. 5C). These findings suggest that the opaque phenotype plays a pivotal role in the survival of *A. baumannii* in its environmental predatory host.

### Complex regulatory network encompassing outer membrane vesicles bound DNA and DNA recombinase RecAB regulate colony switching phenotype

**Fig 6:**
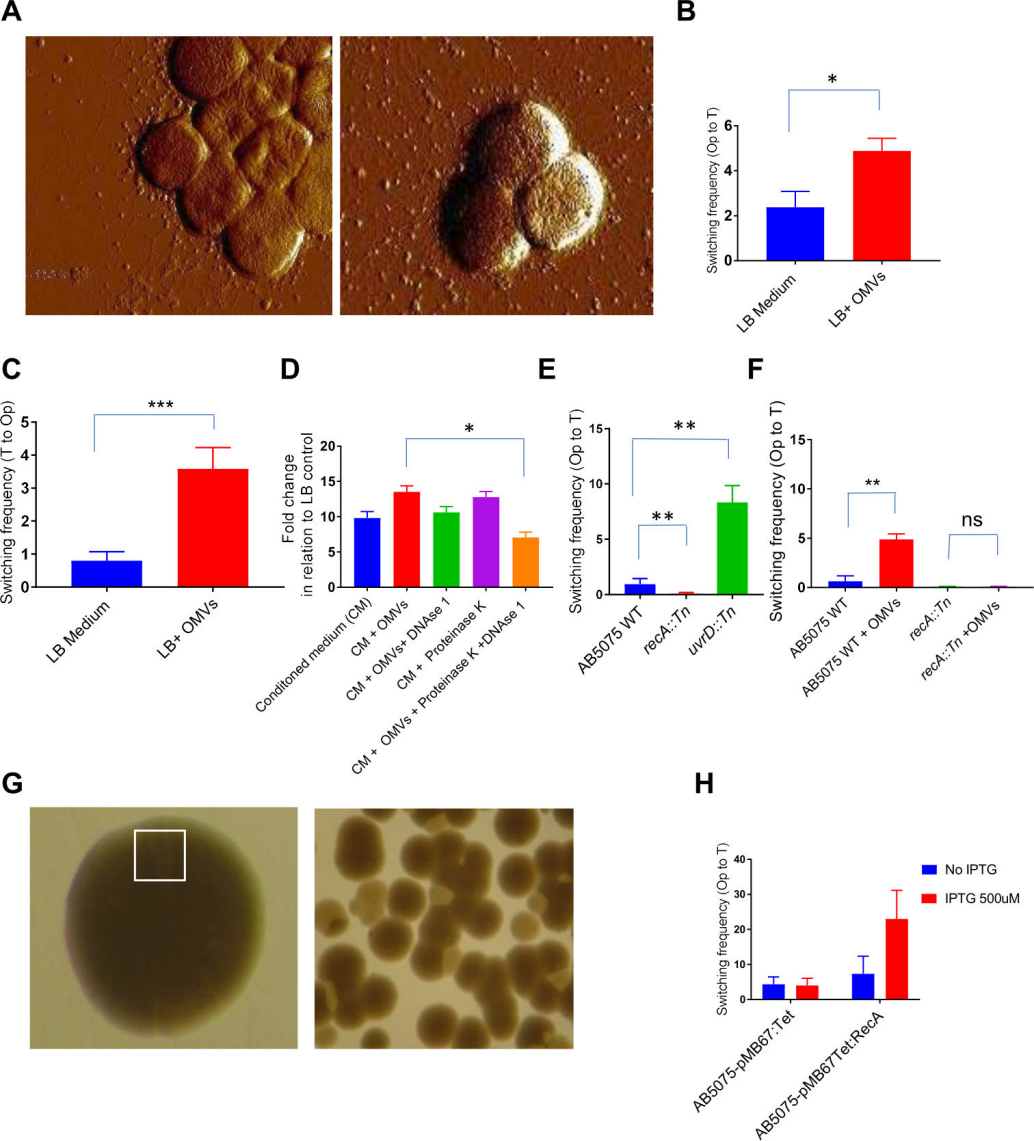
Effect of outer membrane vesicles and OMVs-bound DNA on colony switch phenotype. (**A**) Atomic force microscopic visualization of *A. baumannii* AB5075 opaque (left panel) and translucent (right panel) variants along with secreted vesicles. Bar chart diagrams to illustrate switching frequencies of *A. baumannii* AB5075 from opaque to translucent (**B**) and translucent to opaque (**C**) upon supplementation of MVs prep at a concentration of 100 mg/mL of total protein contents. (**D**) Column bar diagram to illustrate the effect of MVs treated with DNAase I and proteinase K (1U each per 100 mg of total protein at 37°C) on the colony switching phenotype. For enzymatic treatment, OMVs were incubated with DNAse 1 (1U/100 mg of MVs protein content) for 30 min at 37°C. Subsequently, proteinase K (1U/100 mg of MVs protein content were added). The suspension was again incubated for 30 min at 37°C. The enzymes were inactivated by incubation of the suspension at 65°C for 10 min. The values shown in *Y*-axis are relative colony count as compared to average number of translucent colonies in the absence of any treatment. CM; sterile conditioned medium obtained after filtering the overnight culture of *A. baumannii* AB5075OP. (**E**) Column bar diagram to compare frequency of opaque to translucent switch in transposon insertion mutants of *A. baumannii* AB5075 *recA*::Tn and *uvrD*::Tn with wild type (WT). (**F**) Column bar diagram to compare frequency of opaque to translucent switch in *A. baumannii* AB5075 upon overexpression of RecAB from the plasmid. (**G**) A representative image of *A. baumannii* AB5075 transformants of pAT04 (pMBB67EH-RecET: Tet) plasmid; Left panel: opaque colonies with overproduction of RecET harbor multiple translucent sectors as shown with rectangle. Right panel: Bacterial colonies upon the plating of 1/10^5^ dilution of the colony shown in left panel. (**H**) Bar chart diagram to show switching frequency upon over production of RecET in tetracycline plates as compared to vector control.

We have shown previously that *A. baumannii* clinical isolates produce extracellular MVs (7). The number of vesicles secreted by translucent variants appeared to be higher than that of their opaque counterparts. However, atomic force microscopy (AFM) examination of MVs in samples of bacterial cells indicated that the samples from the translucent colony variant contained MVs that were clearly dissociated from the bacterial cells, whereas vesicles appeared to merely remain associated with the bacterial cell surface of the opaque variant (Fig. 6A). We further investigated the impact of secreted MVs on colony switch frequency. The exogenous supplementation of MVs (10 µg/mL) to sterile, conditioned media increased the opaque to translucent switch by twofold and the translucent to opaque switch by threefold (Fig. 6B and C). To investigate whether MVs induce colony switching through their proteomic contents or MVs-bound DNA, the MVs-prep was treated with proteinase K and DNase I. The effect of the treated MVs on switching capacity was then tested. Treatment with proteinase K or DNase I alone did not alter the effect of MVs on colony switching (Fig. 6D). Instead, the treatment of proteinase K along with DNase I reduced the frequency of MV-mediated colony switching. These findings suggest that the DNA contents of MVs protected by the vesicle membrane could play a role in stimulating the colony switching phenomenon. DNA sequencing of the whole DNA content of MVs secreted by *A. baumannii* clinical isolates revealed that MVs contain fragments of random DNA of 100–400 bps lengths, including recombined fragments (Fig. S3).

The colony phase variation is known to be regulated by DNA rearrangement, including DNA recombinase machinery and DNA methylation-associated epigenetic alterations in many bacteria (13, 14). The finding that DNA is one of the stimuli that can promote switching frequency prompted us to investigate the role of DNA repair machinery in colony switching phenotype. In this regard, the colony switching phenotype in AB5075 was tested using transposon insertion mutants of the stress response regulators UvrD helicase (ABUW_2521) and the DNA strand exchange and recombination protein enzyme RecA (ABUW_1739). The *recA::Tn* mutant was not able to switch into the translucent variant, whereas the switching frequency was significantly increased in the *uvrD::Tn* mutant (Fig. 6E). This finding suggests that the DNA recombinase RecA is required for colony switching. However, surprisingly, the UvrD helicase appears to be a negative regulator of the switching phenotype. The switching frequency of the *recA*::Tn mutant was tested in the presence of MVs to investigate if the effect of MVs on colony switching is dependent on RecA. MVs did not trigger the switching of the *recA::Tn* mutant (Fig. 6F). This finding suggests that the effect of MVs on the colony phase variation switch might be dependent on RecA. RecET, a well-known DNA recombinase with respect to activity in *A. baumannii*, was expressed in *A. bau*mannii AB5075 via plasmid pMBB67EH in order to further confirm that DNA recombination machinery is involved in colony phase variation drive. The expression of RecET led to the formation of translucent sectors in opaque colonies (Fig. 6G). The switching frequency of these translucent sectored opaque colonies into translucent colonies was 10-fold higher as compared to opaque AB5075 (Fig. 6H). These findings suggest that a complex regulatory network involving extracellular MVs-bound DNA, stress-inducing stimuli such as tetracycline, and stress response regulators play a role in mediating colony morphotype switching.

## DISCUSSION

Despite the well-established impact of the colony phase variation switch on bacterial lifestyle in *A. baumannii*, the precise molecular mechanism of switching remained a puzzle. Here, we propose that the transfer of DNA fragments associated with membrane vesicles promotes the RecA-mediated colony switching phenotype (Fig. 6). Colony phase variation is known to be regulated by DNA rearrangements, including DNA recombinase machinery and DNA methylation-associated epigenetic alterations in many bacteria (13, 14). Meningococcal DNA recombinase regulates multiple phenotypes associated with phase variation events, such as homologous recombination, pilus antigenic variation, and transformation (15). Here, we find that DNA recombinase and MVs associated DNA fragments play an important role in the mediation of colony phase variation. We hypothesize that DNA recombinase-mediated random DNA rearrangements may be a potential cause of stable colony phase variation. The observation of MV-associated fusion of two distantly located DNA fragments and the efficient ability of MVs to fuse with bacterial cells (Fig. S3) suggest that OMVs may play a role in these types of DNA rearrangements. These findings open a further question, how MVs transport their nucleotide content into cytosol in *A. baumannii*. MVs have the capability to convey information and exert their effects across the cell membrane by either direct binding to surface receptors and fusion with the recipient cell membranes (16). These actions trigger intracellular signaling pathways and facilitate the complete uptake of MV content (17). Nevertheless, it remains unclear which of these mechanisms is responsible for the uptake of MVs containing DNA.

**Fig 7:**
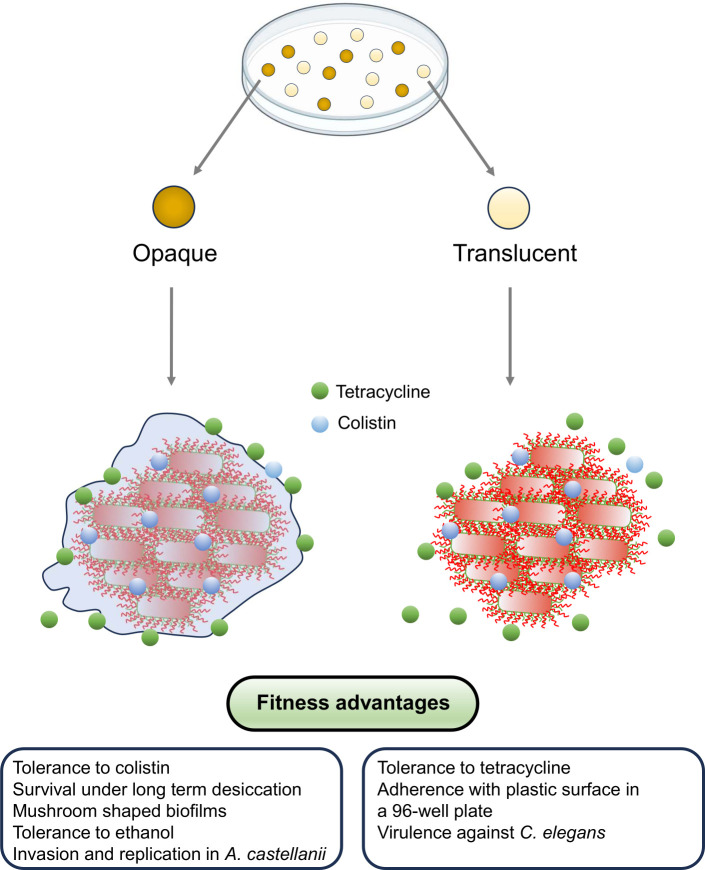
Schematic diagram to summarize fitness advantages of opaque and translucent colony morphotypes of *A. baumannii.* The production of extracellular sheet protects opaque variant from suboptimal concentration of colistin. The fitness advantages here summarized also include findings of our previous study (7).

The opaque morphotype is a well-characterized virulence factor in *A. baumannii* (3) that provides multiple additional fitness to *A. baumannii* (Fig. 7). The opaque colony morphotype also predominates in clinical isolates of *A. baumannii* underscoring its significant contribution to bacterial fitness (7). Furthermore, even other clinically relevant species, such as *Acinetobacter haemolyticus*, exhibit efficient colony morphotype switching their colony morphotype, as demonstrated in our preliminary screening of the colony switching phenotype in some selected *A*cinetobacter species (Fig. S4). Consequently, we propose that the opaque colony feature provides a fitness advantage, enabling pathogenic *Acinetobacter* strains to prevail in causing virulence and survive in stressful environmental conditions.

Colony phase variation switch appears to alter the MIC of colistin. It has been shown using *E*-test that gentamycin, amikacin, and tobramycin also exhibit pronounced alteration in MIC values against opaque and translucent *A. baumannii (3*). However, *E*-test did not detect alteration in MIC of colistin between both types. As the *E*-test is not always suitable to test the MIC of colistin (3), we used microbroth dilution method and growth curve to detect the effect of colony phase variation switch on colistin susceptibility.

We also identified the effect of colony phase variation in the susceptibility to tetracycline (Fig. 2C). However, further investigations are required to identify the mechanism of tetracycline-induced growth suppression but increased MIC of tetracycline in translucent *A. baumannii* AB5075. Since other clinical isolates were intrinsically resistant to tetracycline, we could not test those isolates for response to tetracycline. Tetracycline resistance is typically acquired through the activation of efflux pumps (18, 19). The family of TetR-type transcriptional regulators is redundantly involved in driving this switching through uncharacterized molecular events (3, 6). Strain AB5075 does not possess intrinsic resistance to tetracycline due to the absence of the *tetRA* cassette (20), despite having multiple copies of TetR-type transcriptional regulator-encoding genes. Detailed functional studies of TetR-type transcriptional regulators in response to suboptimal concentrations of tetracycline may elucidate the precise molecular mechanism of the colony phase variation switch phenotype.

Virulent opaque AB5075 formed colistin-tolerant mushroom-shaped biofilm structures in a glass chamber when grown in an artificial urine medium (Fig. 3B and C). This feature was not observed in the avirulent translucent *A. baumannii*. The formation of mushroom-shaped structures by *A. baumannii* strain 17978 in the environmental settings of roller biofilm bioreactors has recently been shown (12). Here, we present evidence of the formation of such types of biofilm structures in more clinically relevant settings. Colistin is the most effective *in vitro* drug against *A. baumannii* isolates causing urinary tract infections; however, it is *in vivo* potency against urinary tract infections is not clearly known (21). Our results suggest that the *in vivo* efficacy of colistin must be carefully considered prior to its use in the treatment of urinary tract infections caused by opaque *A. baumannii*.

Opaque *A. baumannii* is found to be more efficient in invading and surviving inside its environmental host *A. castellanii*, where it resides in *A. baumannii*-containing vacuoles (Fig. 5) despite of having any growth advantage in optimal laboratory conditions (3). The thicker capsule likely facilitates its fitness to adapt to an intracellular environment, as previously shown for the K1 capsule’s pivotal role in the invasion and survival of *E. coli* in *A. castellanii* (22). The expression of extracellular moieties, such as PNAG, may also have a similar protective effect during the interaction of *A. baumannii* with Acanthamoeba, as observed for cellulose synthase and type 1 fimbriae in *Salmonella typhimurium* (23, 24). Since *A. castellanii* shares several environmental niches, such as soil and water reservoirs, with *A. baumannii*, we postulate that it can be an extensive reservoir of this notorious nosocomial pathogen.

Colony phase variation of *A. baumannii* can be considered as an important strategic feature contributing to its fitness and success as a human pathogen. *A. baumannii’s* capacity to transition between distinct colony morphotypes provides it with diverse advantages in a variety of environmental and host contexts, allowing it to adapt and flourish under different circumstances. This adaptability may contribute to the pathogen’s ability to infect healthcare settings and vulnerable individuals. Further studies are required to understand how this bacterium acquired this feature and the precise molecular mechanisms mediating this trait.

## MATERIALS AND METHODS

### Bacterial strains and growth conditions

All the strains and plasmids used in the study are listed in Table S1. All the strains were routinely grown on LB agar plates at 37°C.

### Colony switching assay

The colony phase variation assay was performed as previously described, with slight modifications (5, 7). Briefly, single colonies from the overnight cultures were grown in LB broth to an OD of 1.8. Serial dilutions of the LB broth culture were made, and 100 µL of allocations was re-plated on LB agar plates. After overnight growth, the total number of viable cells per mL was determined. The morphology of opaque or translucent colonies was reviewed by stereo light microscopy. The number of these two-phase variants of colonies was counted only on plates with ≥200 colonies/plate.

### OMV preparation

OMVs were isolated from bacterial culture supernatants as described previously (25). Briefly, 1 L of each bacterial culture, grown in LB broth at 37°C for 16 h, was centrifuged at 5,000 × *g* for 30 min at 4°C. The supernatant was filtered through a 0.2-µm pore size sterile Minisart High Flow syringe filter (Sartorius Stedim) and ultracentrifuged at 100,000 × *g* for 2 h at 4°C in a 45 Ti rotor (Beckman). The vesicle pellet was resuspended in 20 mM Tris-HCl pH 8.0 buffer, and the suspension was used as the crude OMV preparation. The OMV preps were analyzed by SDS-PAGE and by atomic force microscopy. The Bicinchoninic Acid (BCA) Assay kit (Thermo Scientific Pierce, Rockford, IL) was used to measure the samples’ total protein content.

### Atomic force microscopy

*A. baumannii* bacteria and vesicles were imaged by AFM as described previously (26) with slight modifications. Briefly, 10 µL of bacterial samples collected from the logarithmic phase of growth with different serial dilutions were placed onto freshly cleaved mica (Goodfellow Cambridge Ltd., Cambridge, United Kingdom). The samples were blot dried and desiccated prior to imaging. Imaging was done on a Multimode 8 Nanoscope AFM equipment (Digital Instruments, Santa Barbara) using tapping mode TM. A silicon probe was oscillated at its resonant frequency of approximately 300 kHz, selected by the Nanoscope software. Images were collected in the air at a scan rate of 0.8–1.5 Hz, depending on the size of the scan and the number of samples (256 or 512 samples/image). The final images were plane fitted in both axes and presented in a surface plot of the height mode.

### Scanning electron microscopy

For scanning electron microscopy of bacterial colonies, 5 µL of the bacterial suspensions in PBS (OD_600_ of 1), from an overnight plate culture, was spotted on LB agar plates. The plates were incubated at 37°C for 24 h. Colonies were fixed by fuming the plate with fixative (2.5% glutaraldehyde in 0.1 M sodium cacodylate) overnight at 4°C. Pieces of agar containing bacterial colonies were removed, dehydrated in graded series of ethanol, and coated with 5 nm gold/palladium.

For scanning electron microscopy of bacterial pellicles, bacterial strains were grown in glass tubes at 28°C for 72 h. The pellicles formed at air-liquid interface were transferred into glass coverslips. The coverslips and pellicle matrix were incubated in fixative solution (2.5% glutaraldehyde in 0.1 M sodium cacodylate) overnight at 4°C, followed by dehydration in a graded series of ethanol and coating with 5 nm gold/palladium. The bacterial cell morphology was analyzed by a field-emission scanning electron microscope (Carl Zeiss Merlin FESEM) using secondary electron detectors at an accelerating voltage of 4 kV and a probe current of 50–100 pA.

### Live cell confocal microscopic imaging of bacteria grown in flow cell

For analyses of biofilms formed in artificial urine medium, assays were performed in a sterilized six-cell flow chamber (cat: 60606, Ibidi GmbH). Bacterial cells expressing green fluorescent protein through plasmid pwH1266: EGFP grown overnight on LB agar plates supplemented with tetracycline (15 µg/mL) were suspended in PBS to an OD_600_  =  1. From this suspension, 20 µL of bacterial suspension was added to each cell, containing 180 µL of artificial urine medium supplemented with tetracycline (15 µg/mL). Cells were incubated in a moist chamber at 30°C for 72 h. Subsequently, the liquid contents were removed, and cells were washed gently with PBS and visualized under confocal microscopy.

For the colistin tolerance assay, bacterial cells expressing green fluorescent protein through plasmid pWH1266: EGFP grown overnight on LB agar plates supplemented with tetracycline (15 ug/mL) were suspended in PBS to an OD_600_  =  1. From this suspension, 20 µL of bacterial suspension was added to each cell, containing 180 µL of AUM (11) with tetracycline 15 µg/mL. Cells were incubated in a moist chamber at 30°C for 72 h. Subsequently, the liquid contents were removed, and cells were washed gently with PBS two times. The cells were loaded with a mixture of colistin (5 µg/mL) and propidium iodide and incubated at 37°C for 30 min and examined using a Leica SP8 inverted confocal laser system (Leica Microsystems) equipped with an HC PL APO 63×/1.40 oil immersion lens. Images were captured and processed using the LasX (Leica Microsystems) and ImageJ software (27).

### Confocal laser microscopic imaging of biofilm treated with colistin

For the analyses of colistin tolerance at the single-cell level, the assays were performed on a sterilized 18-well glass chamber slide (Ibidi GmbH). Bacterial cells grown overnight on LB agar plates were suspended in PBS to an OD_600_  =  1. From this suspension, 20 µL of bacterial suspension was added to each well, containing 180 µL of LB broth. Chamber slides were incubated in a moist chamber at 30 °C for 72 h. Subsequently, the liquid contents were removed, and the plates were washed gently with PBS. The cells were loaded with colistin 5 U/mL and propidium iodide and incubated at 37°C for 30 min. Components of the biofilm were investigated by staining the bacterial biofilm with Alexa 647-labeled Wheat Germ Agglutinin (Alexa647WGA, 20 µg/mL) and examined using a Leica SP8 inverted confocal laser system (Leica Microsystems) equipped with an HC PL APO 63×/1.40 oil immersion lens. Images were captured and processed using the LasX (Leica Microsystems) and ImageJ software (27).

### MIC determination of colistin and tetracycline

The minimal inhibitory concentration of colistin was determined by the microbroth dilution method. The base material, colistin (Glaxosmith Kline Pharmaceuticals) was prepared in water and tetracycline in 70% ethanol and stored at −20°C for 1 week. A bacterial inoculum equivalent to 0.5 McFarland (5 × 10^8^) was prepared and diluted 1:10 to achieve the final inoculum of 5 × 10^7^ CFU/mL. The concentration range of the antibiotics to be tested was 0.125–256 µg in MHB using 96-well plates. The plates were incubated for 24 h at 37°C and the lowest concentration at which bacterial growth was completely inhibited was noted and declared the MIC (CLSI 2020). The MIC results were read and interpreted according to the CLSI 2019 breakpoints for *A. baumannii*.

### Fluorescence-based measurement of extracellular polysaccharide moieties

Extracellular polysaccharide moieties were measured based on their binding with Alexa647-labeled WGA. For the WGA binding measurement assay, bacterial strains were grown on LB agar plates overnight at 37°C. The cells grown within opaque or translucent colonies were normalized to OD_600_ = 0.1 suspension with PBS. The normalized cell suspensions (100 µL) were treated with WGA (20 µg/mL) for 1 h at 37°C in microfuge tubes. The tubes were centrifuged for 5 min at 5,000 × *g*. The supernatant was discarded, and the pelleted bacteria were resuspended with 100 µL of PBS. The centrifugation step was repeated two times. The fluorescence of the contents was measured using a fluorescent plate reader.

### Assay to monitor long-term survival under desiccation

For the long-term survival assay, bacterial strains were grown on LB agar plates overnight at 37°C. The bacteria grown from opaque or translucent colonies were adjusted to OD_600_ = 0.1 and washed three times with PBS and pelleted by centrifugation. An inoculum of 100 μlwas spotted on each well of the 96-well polystyrene plates. The contents were let to dry through evaporation, and plates were incubated in the dark with sampling to monitor viable bacterial counts periodically for up to 150 days.

### Bacterial competition assay

For the competition tests between *A. baumannii* and *E. coli*, bacterial strains were grown on LB agar plates overnight. Colonies from each sample were resuspended in PBS and adjusted to OD_600_ = 0.1. The normalized cell suspensions of *E. coli* and *A. baumannii* samples at a ratio of 1:1 were mixed and serially diluted to 6- to 10-fold dilutions. A drop of 10 µL volume from each of the serial dilutions was spotted on LB agar plates supplemented with IPTG, carbenicillin, and X-gal. The plates were incubated at 37°C for 24 h. The number of CFU corresponding to *E. coli* (blue colonies) and to *A. baumannii* (opaque or translucent white colonies) were counted from the spots where individual bacterial colonies were countable. The representative spots from zeroth dilution (left panel) and fourth dilution (right panel) are shown in Fig. 4E. Each experiment was repeated three times.

### Ethanol tolerance assay

For the ethanol tolerance assay, bacterial strains were grown on LB agar plates over night at 37°C. The cells grown within opaque or translucent colonies were normalized to OD_600_ = 0.1 with PBS. The normalized cells (100 µL) were treated with 5% of ethanol at 25°C in microfuge tubes. The tubes were centrifuged for 5 min at 5,000 × *g*. The supernatant was discarded, and the pellet was resuspended with 100 µL of PBS. The resuspension was serially diluted to 6- to 10-fold, dilutions. A drop of 10 µL volume from each of the serial dilutions was spotted on LB agar plates. The plates were incubated at 37°C for 24 h. The number of CFU was counted from the spots where individual bacteria colonies were countable. Each experiment was repeated five times.

### Confocal microscopy and CFU counts for interaction of *A. baumannii* with *A. castellanii*

To determine the ability of opaque and the translucent variants of *A. baumannii* AB5075 containing pWH1266:EGFP plasmid to invade or be phagocytosed by *A. castellanii*, an invasion assay was performed. Briefly, *A. castellanii* (1 × 10^4^) was seeded in PYG media in an 18-well coverslip bottom glass chamber slides, overnight at 25°C. The next day, *A. castellanii* was washed with nine salt solution (NSS). The NSS was prepared by dissolving 17.6 g NaCl, 1.47 g Na_2_SO_4_, 0.08 g NaHCO_3_, 0.25 g KCl, 0.04 g KBr, 1.87 g MgCl_2_·6H_2_O, 0.45 g CaCl_2_·2H_2_O, 0.01 g SrCl_2_·6H_2_O, and 0.01 g H_3_BO_3_ in 1 L of distilled water. *A. castellanii* was infected with variants of *A. baumannii* AB5075 at an multiplicity of infection (MOI) of 200 for 24 h at 25°C. *A. castellanii* infected with variants of *A. baumannii* AB5075 containing pWH1266:EGFP plasmid were visualized live using confocal microscope. In parallel, *A. castellanii* infected with *A. baumannii* AB5075 opaque containing pWH1266:EGFP plasmid for 24 h, followed by fixation and staining with Hoechst 33342, and actin using Phalloidin-Alexa596 for 30 min. *A. castellanii* infected with bacteria were imaged by a Leica SP8 inverted confocal laser system (Leica Microsystems) equipped with an HC PL APO 63×/1.40 oil immersion lens. Images were captured and processed using the LasX (Leica Microsystems).

For colony-forming unit (CFU) counts, *A. castellanii* (5 × 10^3^) was infected in nine salt solution with variants of *A. baumannii* AB5075 at an MOI of 200 for 48 h at 25°C. For competition experiment, *A. castellanii* was infected with a mixture of opaque (MOI: 100) and translucent (MOI: 100) variants of *A. baumannii* AB5075 for 48 h at 25°C. For count of total bacteria, the intracellular bacteria were released by lysis of *A. castellanii* with TritonX-100 (0.5%) at 37°C for 30 min, followed by serial dilution and spreading on LA plates. Serial dilutions of the collected bacteria were made, followed by their spreading on LA plates.

### Whole-genome sequencing of OMVs bound DNA

The DNA of the vesicles was extracted using a total DNA isolation kit (Thermo Scientific). The whole DNA sequencing was performed using the MiSeq Desktop Sequencer and MiSeq Reagent Kit v3 (Illumina, San Diego, CA, USA). DNA preparation, library construction, and genome sequencing were done according to the manufacturer’s instructions. Sequence data were assembled and analyzed using the CLC genomics workbench (v7.0.4; CLC bio, Aarhus, Denmark). DNA sequences were deposited to gene bank. Accession numbers are enlisted in Fig. S3A.

### Statistical analysis

The statistical significance of the difference was measured by a nonparametric *t* test using Graph Pad Prism 7. A single * indicates *P* ≤ 0.05, ** indicates *P* ≤ 0.01, *** indicates *P* < 0.001, and ns = non-significant. The difference was compared to the respective opaque variant, otherwise indicated by horizontal bars above column bars.

## SUPPLEMENTAL MATERIAL

Supplemental figures spectrum.02956-23-s0001.pdf Fig. S1 to S4.

Table S1 spectrum.02956-23-s0002.docx Strains used in the study.

ASM does not own the copyrights to Supplemental Material that may be linked to, or accessed through, an article. The authors have granted ASM a non-exclusive, world-wide license to publish the Supplemental Material files. Please contact the corresponding author directly for reuse.
