# Supplementary material for: Colony phase variation switch modulates antimicrobial tolerance and biofilm formation in Acinetobacter baumannii
Source: Microbiol Spectr. 2024 Jan 11;12(2):e02956-23. doi: 10.1128/spectrum.02956-23 (PMC10845969; doi:10.1128/spectrum.02956-23)
Supplement: Supplemental figures — Fig. S1 to S4. [file spectrum.02956-23-s0001.pdf]

Supplementary Figures

Colony phase variation switch modulates antimicrobial tolerance and biofilm formation in *Acinetobacter baumannii.*

Fizza Mushtaq[1,2, #], Aftab Nadeem[1, #], Abdelbasset Yabrag [1], Anju Bala[1], Nabil Karah[1], Nikola Zlatkov[1], Sun Nyunt Wai[1,3], Bernt Eric Uhlin[1], Irfan Ahmad[1, 2, *]

[1]Department of Molecular Biology and Umeå Centre for Microbial Research (UCMR), Umeå University, SE-90187 Umeå, Sweden

[2]Institute of Biomedical and Allied Health Sciences, University of Health Sciences, Lahore, Pakistan

[3]The Laboratory for Molecular Infection Medicine Sweden (MIMS), Umeå University, SE-90187 Umeå, Sweden

[#]Equal contributions

[*]Correspondence:

Irfan Ahmad

Irfan.ahmad@umu.se

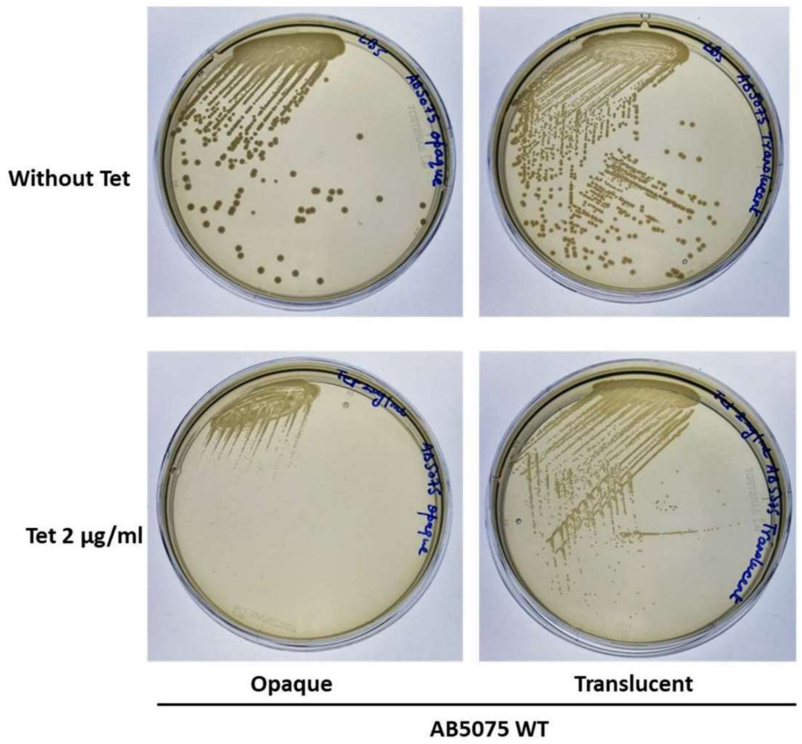

Figure S1: Bacterial colonies after the growth of *A. baumannii* AB5075 opaque and translucent variants on LB agar in the presence and absence of tetracycline. The plates were incubated 24 hours at 37ºC.

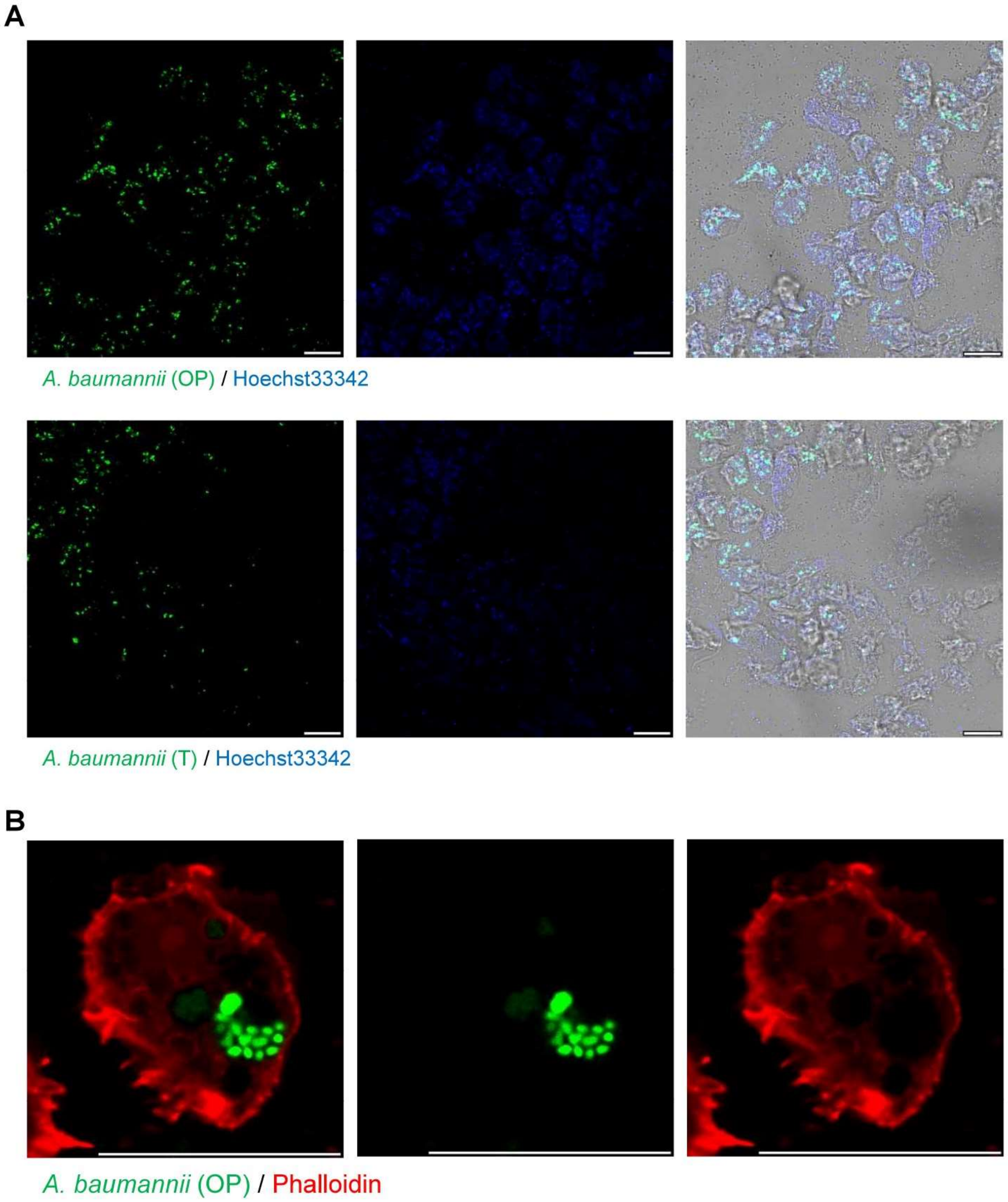

Figure S2: Invasion of *A. castellanii* by opaque and translucent *A. baumannii*. (A) Confocal microscopy of *A. castellanii* invaded by opaque and translucent variant of EGFP-*A. baumannii* AB5075 (green). Cells were counterstained with Hoechst 33342 (blue) Scale bars = 20 μm. (B) Confocal micrograph for visualization of single section of *A. castellanii*. The image shows presence of opaque *A. baumannii* multicellular communities (green), present within a vacuole of *A. castellanii*, co-stained with actin binding fluorescent dye Phalloidin594 (red)**.** Scale bars = 20 μm

A

OMVs *A. baumannii* S02	Length of fragment	Encoded proteins	GC Contents	Gene bank Accession number
Contig 1	306 bps	Hypothetical protein	40	OR677839
Contig 2	249 bps	Hyothetical protein	43	OR677840
Contig 3	300bps	Lysophospholipase	40	OR677841
Contig 4	264bps	mechanosensitive ion channel family protein DUF2339 domain protein	39	OR677842
**OMVs *A. baumannii* S14**				
Contig 1	570 bps	Hypothetical protein Full ORF along promoter	39	OR661843
Contig 2	228	Membrane protein	45	OR661844
Contig 3	234	SulP family inorganic anion transporter	48	OR661845
Contig 4	215	Hypothetical protein	42	OR661846
Contig 5	328	HlyD family type I secretion periplasmic adaptor ATP binding protein	41	OR661847
**OMVs *A. baumannii* S17**				
Contig 1	292	1983 DUF domain	40	OR677835
Contig 2	583	ATP synthase	44	OR677836
Contig 3	318	Hypothetical	38	OR677837
Contig 4	232	SfnB family sulphur acquision oxidoreductase	43	OR677838
**OMVs *A. baumannii* S23**				
Contig 1	233	ABUW 0874 Dihydrolipoamide dehydrogenase	38	OR677843
Contig 2	251	Sulp family inorganic ion trtansporter and 16 sRNA	38	OR677844
Contig 3	244	ABUW_893 carbamoyl phosphate synthase	45	OR677845
Contig 4	259	ABUW1043 Asparate aminotransferase	44	OR677845
Contig 5	269	Anthranilate 1,2 dioxygenase	43	OR677847
Contig 6	203	NADP dependent isocitrate dehydrogenase	47	OR677848
Contig 7	223	ABUW_2764 Monooxygenase	40	OR677849
Contig 8		DDE type integrase		OR677850

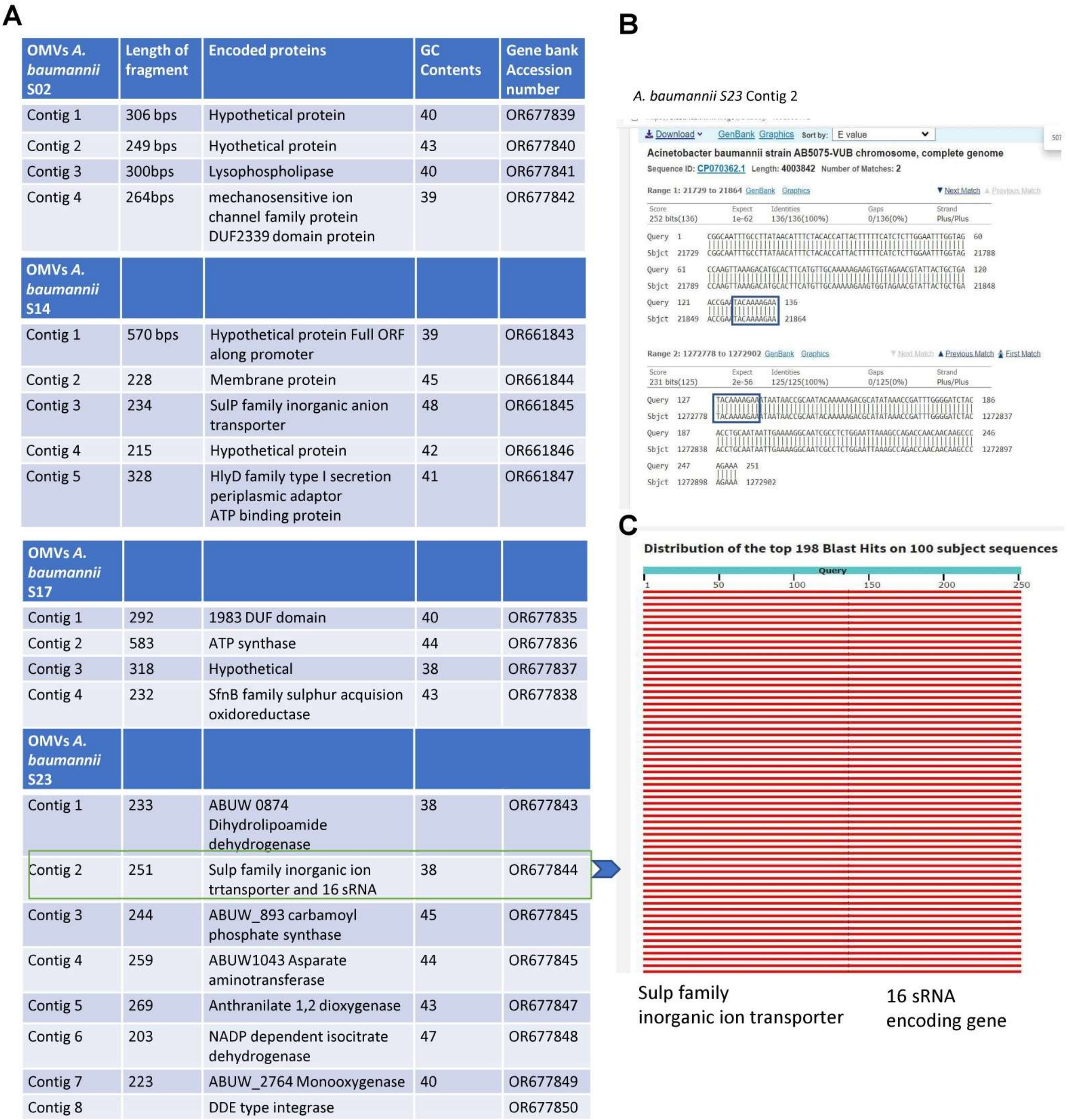

Figure S3: Genetic content of MVs bound DNA identified through whole genome sequencing of DNA isolated from MVs

Left panel: Summary of MVs bound DNA content in clinical isolates; Right Panel: DNA sequence of Contig 4 harbouring DNA fusion in partial sequence of genes encoding Sulp family inorganic transporter and 16sRNA. The fusion occurs through a 10bp long homologues sequence common in both genes shown within rectangle. The sequence formed upon the fusion of both fragments is not found in any sequenced genome of *A. baumannii.*

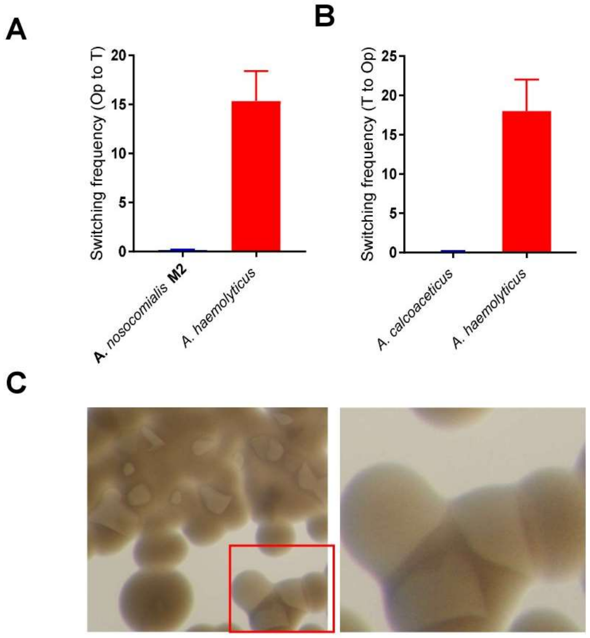

Figure S4: (Upper panel) Switching frequency of *Acinetobacter* species. (Lowe Panel) Stereomicroscopic visualization *A. haemolyticus* colonies on agar plates after 48 hours of incubation at 37ºC.
