# Supplementary material for: Colony phase variation switch modulates antimicrobial tolerance and biofilm formation in Acinetobacter baumannii
Source: Microbiol Spectr. 2024 Jan 11;12(2):e02956-23. doi: 10.1128/spectrum.02956-23 (PMC10845969; doi:10.1128/spectrum.02956-23)
Supplement: Table S1 — Strains used in the study. [file spectrum.02956-23-s0002.docx]

**Table S1: Strains used in the study**

| **Strain ID** | **Genotype** | **Reference** |
| --- | --- | --- |
| *Acinetobacter baumannii 17978* | Wild type | ATCC17978 |
| *Acinetobacter baumannii AB5075* | Wild type | (1) |
| *Ab-pak-Lah-14* | Clinical isolate | (2, 3) |
| *Ab-pak-Lah-7* | Clinical isolate | (2, 3) |
| *Ab-pak-pesh-37* | Clinical isolate | (2, 3) |
| *Ab-pak-pesh-22* | Clinical isolate | (2, 3) |
| *recA::Tn* | AB5075 Transposon insertion mutant in locus encoding RecA *(*ABUW1748) | (1) |
| *uvrD::Tn* | AB5075 Transposon insertion mutant in in locus encoding RecA *(*ABUW_2521) | (1) |

**References**

1. Gallagher LA, Ramage E, Weiss EJ, Radey M, Hayden HS, Held KG, Huse HK, Zurawski DV, Brittnacher MJ, Manoil C. 2015. Resources for Genetic and Genomic Analysis of Emerging Pathogen Acinetobacter baumannii. J Bacteriol 197:2027-35.

2. Ahmad I, Karah N, Nadeem A, Wai SN, Uhlin BE. 2019. Analysis of colony phase variation switch in Acinetobacter baumannii clinical isolates. PLoS One 14:e0210082.

3. Karah N, Khalid F, Wai SN, Uhlin BE, Ahmad I. 2020. Molecular epidemiology and antimicrobial resistance features of Acinetobacter baumannii clinical isolates from Pakistan. Ann Clin Microbiol Antimicrob 19:2.
